# Evaluation of a Multilocus Sequence Typing (MLST) scheme for *Leishmania* (*Viannia*) *braziliensis* and *Leishmania* (*Viannia*) *panamensis* in Colombia

**DOI:** 10.1186/s13071-017-2175-8

**Published:** 2017-05-12

**Authors:** Giovanny Herrera, Carolina Hernández, Martha S. Ayala, Carolina Flórez, Aníbal A. Teherán, Juan David Ramírez

**Affiliations:** 1 Grupo de Investigaciones Microbiológicas-UR (GIMUR), Programa de Biología, Facultad de Ciencias Naturales y Matemáticas, Universidad del Rosario, Carrera 24 No. 63C-69, Bogotá, Colombia; 2 Grupo de Parasitología, Instituto Nacional de Salud, Avenida Calle 26 #51-20, Bogotá, Colombia; 3 Residente Medicina de Emergencias, Escuela de Medicina y Ciencias de la Salud, Universidad del Rosario, Carrera 24 No. 63C-69, Bogotá, Colombia

**Keywords:** *Leishmania*, MLST, DST, Cutaneous leishmaniasis, Genetic diversity

## Abstract

**Background:**

Leishmaniases are parasitic vector-borne diseases affecting more than 12 million people in 98 countries. In Colombia, leishmaniasis is widespread and the most common clinical manifestation is cutaneous, mainly caused by *L. panamensis* and *L. braziliensis*. Currently, the genetic diversity of these species in Colombia is unknown. To address this, we applied molecular techniques for their characterization, using multilocus sequence typing (MLST) to explore the genetic variability and phylodynamics of the disease.

**Methods:**

Seven previously described genetic markers were selected highlighting the implementation of a mitochondrial marker. Markers were applied to 163 samples from isolates obtained between 1980 and 2001.

**Results:**

The identification of the samples showed an excellent correlation with typing tests previously applied (MLEE, monoclonal antibodies). Isolates of *L. braziliensis* showed greater genetic diversity than *L. panamensis*, and a greater number of diploid sequence types (DSTs). In addition, the geographical distribution of DSTs for each species were obtained through georeferencing maps.

**Conclusions:**

To our knowldge, this study represents the first description of the genetic variability of *L. panamensis* in Colombia and South America, and is the first to propose a scheme of MLST for epidemiological surveillance of leishmaniasis in the country.

**Electronic supplementary material:**

The online version of this article (doi:10.1186/s13071-017-2175-8) contains supplementary material, which is available to authorized users.

## Background

Leishmaniases are a group of diseases caused by *Leishmania* parasites and transmitted to humans and other mammals by the bite of psychodid insects [1]. Currently, leishmaniases are ranked among the most neglected diseases constituting a serious public health problem worldwide because of high morbidity and the fact that the areas where most cases occur are usually rural or suburban, hindering the diagnosis and treatment of infected patients. The clinical presentations of the disease are cutaneous leishmaniasis (CL), mucocutaneous leishmaniasis (MCL) and visceral leishmaniasis (VL) [1, 2]. These pathologies affect more than 12 million people in 98 countries and is estimated that 350 million individuals are at risk of acquiring the infection, with an incidence of 1.3 million of new cases per year [3, 4]. In the Americas, CL and MCL are endemic in 18 countries with a distribution of cases from Mexico to Argentina, where Brazil, Colombia and Peru report the highest percentage of cases [5]. In Colombia, it is estimated that the population at risk of infection is 12,277,606 people for CL and 6,795,047 people for MCL respectively [4].

The genus *Leishmania* includes more than 20 species grouped in complexes constantly under review and strong debate [6]. In Colombia, *L. panamensis*, *L. braziliensis*, *L. lainsoni*, *L. guyanensis*, *L. infantum chagasi*, *L. equatoriensis*, *L. mexicana*, *L. amazonensis* and *L. colombiensis* have been reported, with *L. panamensis* and *L. braziliensis* as the most prevalent and mainly associated to CL [7, 8]. However, many studies in the country do not report the species involved in the transmission, which has hindered the association with the clinical presentation, profile of susceptibility to treatment and epidemiological management of outbreaks [9–11]. In this sense, molecular biology tools have been shown to be very useful, not only for diagnosis, but also for the identification of species [12]. In addition to multilocus enzyme electrophoresis (MLEE), considered the gold standard test [13], other techniques such as multilocus sequence typing (MLST) have been used as a valuable tool for the characterization of the *L. donovani* complex [14, 15]. However, the lack of databases for *Leishmania*, added to the low number of typed strains and the lack of consensus on the markers has hindered the implementation of this technique as a reference method. Parallel to MLST, multilocus sequence analysis (MLSA) has been employed in the study of *Leishmania braziliensis* for analysis of genetic diversity and outbreaks surveillance [16, 17]. This methodology, widely used for several pathogens [18–20], has become an important tool for the study of *Leishmania* [16, 17]. Nevertheless, few studies have been reported in South America (Brazil and Argentina). In Colombia, no MLST or MLSA have been applied resulting in the absence of information concerning the transmission dynamics of leishmaniasis in the country.

Therefore, the aims of this study were to standardize and evaluate an MLST scheme based on seven markers in *L.* (*Viannia*) *panamensis* and *L.* (*Viannia*) *braziliensis* strains from different geographical regions of Colombia. To determine the circulating diploid sequence types (DSTs) and strengthen the epidemiological surveillance in the country; and finally, to provide further data for the understanding of the genetic diversity of *Leishmania* parasites in South America.

## Methods

### Sampling

In this study, we included a total of 163 isolates of *L. panamensis* (105 isolates) and *L. braziliensis* (58 isolates) from human cutaneous leishmaniasis cases obtained by the National Institute of Health of Colombia (NIHC) as part of epidemiological surveillance between 1980 and 2001. Isolates were retrieved from patients from 20 departments (Antioquia, Bolivar, Boyaca, Caldas, Caquetá, Casanare, Chocó, Córdoba, Cundinamarca, Guainía, Guaviare, Magdalena, Meta, Norte de Santander, Putumayo, Risaralda, Santander, Sucre, Tolima and Vichada).

### Parasite isolation, DNA extraction and typing

Punch biopsies were triturated in sterile Ten Broeck homogenizers containing phosphate buffered saline (PBS), gentamicin (40 μg/ml), and 5-fluorocytosine (500 μg/ml). The resultant tissue suspension was inoculated directly into two tubes of NNN medium. *Leishmania panamensis* and *L. braziliensis* species identification was performed for each isolate by direct Sanger sequencing of cytochrome b, heat shock protein 70 (HSP70) and MLEE as described by Ramirez et al. [7]*.* DNA extraction was performed using the Quick-DNA™ Universal Kit (Zymo Research, Orange, CA, USA) following the manufacturer's protocol.

### Marker selection, amplification by PCR and sequencing

Amplification was performed based on specific primers for gene fragments coding for metabolic enzymes such as glucose 6-phosphate dehydrogenase (*g6pdh*, EC 1.1.1.49), phosphoglucomutase (*pgm*, EC 5.4.2.2), mannose phosphate isomerase (*mpi*, EC 5.3.1.8), alanino-transferase (*alat*, EC 2.6.1.2), aspartate aminotransferase (*asat*, EC 2.6.1.1), isocitrate dehydrogenase (*icd*, EC 1.1.1.42) and cytochrome *b* (*cytb*) molecule as previously reported [15, 16, 21] (Table 1). The PCR reactions were performed in a final volume of 25 μl containing 12.5 μl GoTaq® Green Master Mix (Promega, Madison, WI, USA), 0.5 μM of each primer and 1.25 μl of DNA (<250 ng). Amplification was conducted in a Thermal Cycler S1000™ (Bio-Rad Laboratories, Inc., Hercules, CA, USA) with an initial denaturation at 95 °C for 5 min, followed by 40 cycles of denaturation at 95 °C for 1 min, annealing at 60 °C for 1 min and extension at 72 °C for 1 min. Finally, an extension at 72 °C was performed for 10 min. Amplification and amplicon size was verified by electrophoresis on agarose gel stained with SYBR Safe DNA Gel Stain (Life Technologies, Carlsband, CA, USA) and a molecular weight marker (Promega) adding 2 μl of each PCR product. The remaining 23 μl were purified using the PCR kit ExoSAP-IT® Product Cleanup (Affymetrix, Santa Clara, CA, USA). Sequencing of both strands was carried out in the Unit of Sequencing and Genomic Analysis of the National Institute of Health, Colombia (NIHC) using the Big Dye Terminator v3.1 kit and ABI 3130 Genetic Analyzer (Applied Biosystems, California, USA).

**Table 1:** List of primers and gene markers used in this study as MLST scheme

Target gene	Enzyme entry	Chromosomal location	Gene length (bp)	Amplicon size (bp)	Primer sequence	Reference
Glucose-6-phosphate dehydrogenase (*g6pdh*)	EC 1.1.1.49	20 and 34a^a^	1,686	881	Fw: ATGGAAGCGTGTGATCGAAT Rv: GGCTCAACACACTTCAGCAA	[16]
Phosphoglucomutase (*pgm*)	EC 5.4.2.2	21	1,770	529	Fw: CAGAGAAGCTGACGTCCCAG Rv: GACGGGTTCACGAAGAAGCG	[21]
Mannose phosphate isomerase (*mpi*)	EC 5.3.1.8	32	1,287	681	Fw: GGCAAGATGTATGCGGAGTT Rv: CTCCCCAGGAACCATCTGTA	[16]
Alanine aminotransferase (*alat*)	EC 2.6.1.21	12	1,494	589	Fw: GTGTGCATCAACCCMGGGAA Rv: CGTTCAGCTCCTCGTTCCGC	[21]
Aspartate aminotransferase (*asat*)	EC 2.6.1.1	24	1,169	684	Fw: ACGAGCGCCGTCCGYAA Rv: TTCCYMCATCCACCAAGC	[15]
Isocitrate dehydrogenase (*icd*)	EC 1.1.1.42	33	1,278	1,022	Fw: GAATCGGGAAGGAGATCACA Rv: CATCATAGCCCCAGAGAGGA	[16]
Cytochrome *b* (*cytb*)		Maxicircle		618	Fw: AGCGGAGAGRARAGAAAAGG Rv: GYTCRCAATAAAATGCAAATC	

^a^ *g6pdh* gene is present on chromosomes 20 and 34a only in *L.* (*V) braziliensis*; it is present on chromosome 20 in *L.* (*V*) *panamensis* uniquely

### Sequence assembly and genetic diversity indices

The assembly of the consensus sequences using the forward and reverse reads, alignment and sequence editing was performed using MEGA 5.0 [22]. Additionally, electropherograms were revised in order to detect ambiguous sites (heterozygosity) evidenced by two overlapped peaks in one position. Reconstruction of haplotypes was performed using the algorithm PHASE, through DnaSP v.5.0 [23]. The indices of genetic diversity of the sequences (total number of mutations Eta, haplotype diversity Hd, genetic diversity ɵ and π and the rate of synonymous and non-synonymous substitutions dN/dS) were calculated for each marker, each species and by geographical region (Andean, Orinoquia, Caribbean, Pacific and Amazon), using DnaSP v.5.0 [23]. Nucleotide diversity (π) is defined as the average number of nucleotide differences per site between two sequences. The θ index is defined as an indicator of mutation rate per nucleotide site per generation. Eta (h) is defined as the total number of mutations and S the number of segregating (polymorphic) sites. Comparison charts of haplotype diversity for each gene were constructed for each species using Microsoft Excel 2013. Additionally, using the same tool, sample distributions were constructed by geographical region.

### Allelic profile coding, geographical and temporal distribution of DSTs

MLSTest (https://mlstest.codeplex.com/) bioinformatic tool was used for DST assignment, based on the allelic profile of each sample using the seven gene fragments of the MLST scheme herein proposed. The DST is defined as the combination of the alleles from the different gene fragments used for an MLST scheme. Similarly, the number of alleles and polymorphic nucleotide numbers were calculated [24]. In addition, a map of DSTs distribution of alleles for each species, based on the georeferenced coordinates of each of the samples was constructed for *L. panamensis* and *L. braziliensis* using the ArcGIS10.3 program (http://www.esri.com/ArcGIS10.3). Finally, we assessed the temporal variation of the different DSTs for each species across the 21 years of sampling.

### Phylogenetic analysis and clonal complexes determination

The final set of sequences from each individual gene fragment and the concatenated alignment were evaluated in ModelTest 3.7 where the most appropriate evolutionary model was selected based on the AIC (Akaike information criterion). A maximum composite likelihood (MCL) analysis using a Tamura-3 parameter model and the Neighbour-Joining algorithm was run in RAxML 7.2.5 on the CIPRES project (Cyberinfrastructure for Phylogenetic Research) portal 2.0 servers. To evaluate the robustness of the nodes in the resulting phylogenetic trees, 1,000 bootstrap replicates were performed. The final trees were rooted with sequences for *Leishmania donovani*, *Leishmania guyanensis*, *Leishmania tropica*, *Leishmania major*, *Leishmania amazonensis* and *Leishmania mexicana*, retrieved from TriTrypDB. DSTs obtained based on the sequences of the seven genes for each species were subjected to eBURST program (http://eburst.mlst.net) to presumably determine the evolutionary relationships among the samples analyzed. eBURST predicts descent from a genotype "founder" for all other genotypes, establishing the groups of related strains which share certain number of identical alleles with other members (Clonal Complex).

### Assesment of clonality *via* allelic plot

The population genetics of *Leishmania* is complex. Therefore, we decided to calculate the rate of topology incongruence as a measure of clonality *vs* sexuality across the seven markers employed. We constructed MCL phylogenetic trees from each gene fragment and the allelic diversity was measured in terms of ‘allelic types’ supported as nodes with bootstrap values over 80% and colored appropriately depending on the allelic type within *L. panamensis* (red) and *L. braziliensis* (blue); this allelic diversity patterns was used to construct an allelic plot to determine the extent of allelic repertoire exchange at inter and intra species level. Finally, based on the combination of different shades of blue and/or red (different number of alleles supported by bootstrap values equal to or over 80%), the number of multilocus genotypes (MLG) were calculated manually. Here MLGs are defined as the combination of statistically supported alleles based on phylogenetic reconstruction using maximum likelihood estimations.

## Results

### Genetic diversity of the samples analyzed

The seven markers evaluated in the present study were successfully amplified in all the samples tested. Genetic diversity calculations showed a greater degree of intraspecific variation in *L. braziliensis* than in *L. panamensis*. However, out of the seven markers evaluated only cytochrome *b* showed greater diversity in *L. panamensis* than in *L. braziliensis* (Table 2). Nucleotide diversity indices π and θ in *L. panamensis* were on average 0.016499 and 0.00815, respectively, while in *L. braziliensis* were 0.02452 and 0.03337, respectively. Fragments of the tested genes showed between 6 and 80 polymorphic sites in *L. panamensis* (80 only in *cytb*) and between 5 and 21 polymorphic sites in *L. braziliensis*. On average, the ratio of the rate of nonsynonymous and synonymous substitutions was higher in *L. panamensis* (dN/dS = 0.1705) than in *L. braziliensis* (dN/dS = 0.1261). The *pgm* and *alat* genes in *L. panamensis* showed the highest values of haplotype diversity, followed by *asat* and *g6pdh* genes, whereas genes in *L. braziliensis* showed more haplotype diversity across *mpi*, *asat* and *cytb* (Additional file 1: Figure S1).

**Table 2:** Genetic diversity indexes of *L. panamensis* and *L. braziliensis* isolates used in this study

Species	Marker	N	S	Eta	Hd	π^a^	θ^b^	dN/dS
*L. panamensis*	*pgm*	105	10	7	0.234	0.00245	0.00422	0.1454
	*asat*	105	6	6	0.145	0.00057	0.00322	0.1745
	*g6pdh*	105	6	6	0.180	0.00078	0.00211	0.1276
	*mpi*	105	7	8	0.113	0.04292	0.05469	0.2346
	*icd*	105	10	8	0.111	0.00067	0.00422	0.0124
	*alat*	105	11	10	0.234	0.0066	0.00722	0.2345
	*cytb*	105	80	85	0.123	0.00304	0.03981	0.2371
*L. braziliensis*	*pgm*	58	13	10	0.324	0.02343	0.04123	0.1352
	*asat*	58	11	7	0.413	0.01509	0.03086	0.1142
	*g6pdh*	58	15	11	0.319	0.01008	0.02003	0.1174
	*mpi*	58	21	13	0.496	0.06271	0.06667	0.1376
	*icd*	58	14	10	0.331	0.02519	0.03113	0.1238
	*alat*	58	17	9	0.223	0.03412	0.04126	0.1927
	*cytb*	58	5	5	0.390	0.00104	0.00243	0.1282

*Abbreviations*: *N* number of sequences, *S* number of polymorphic sites, *Eta* total number of mutations, *Hd* haplotype diversity

^a^π is an index of nucleotide diversity. This measure is defined as the average number of nucleotide differences per site between any two DNA sequences chosen randomly from the sample population

^b^The mutation parameter (θ) is defined as 4 Nm for autosomal loci of diploid organisms, where N is the effective population size (diploid individuals) and m is the neutral mutation rate (per gene or per base pair) per generation

### Genetic diversity by geographical regions

**Fig. 1:**
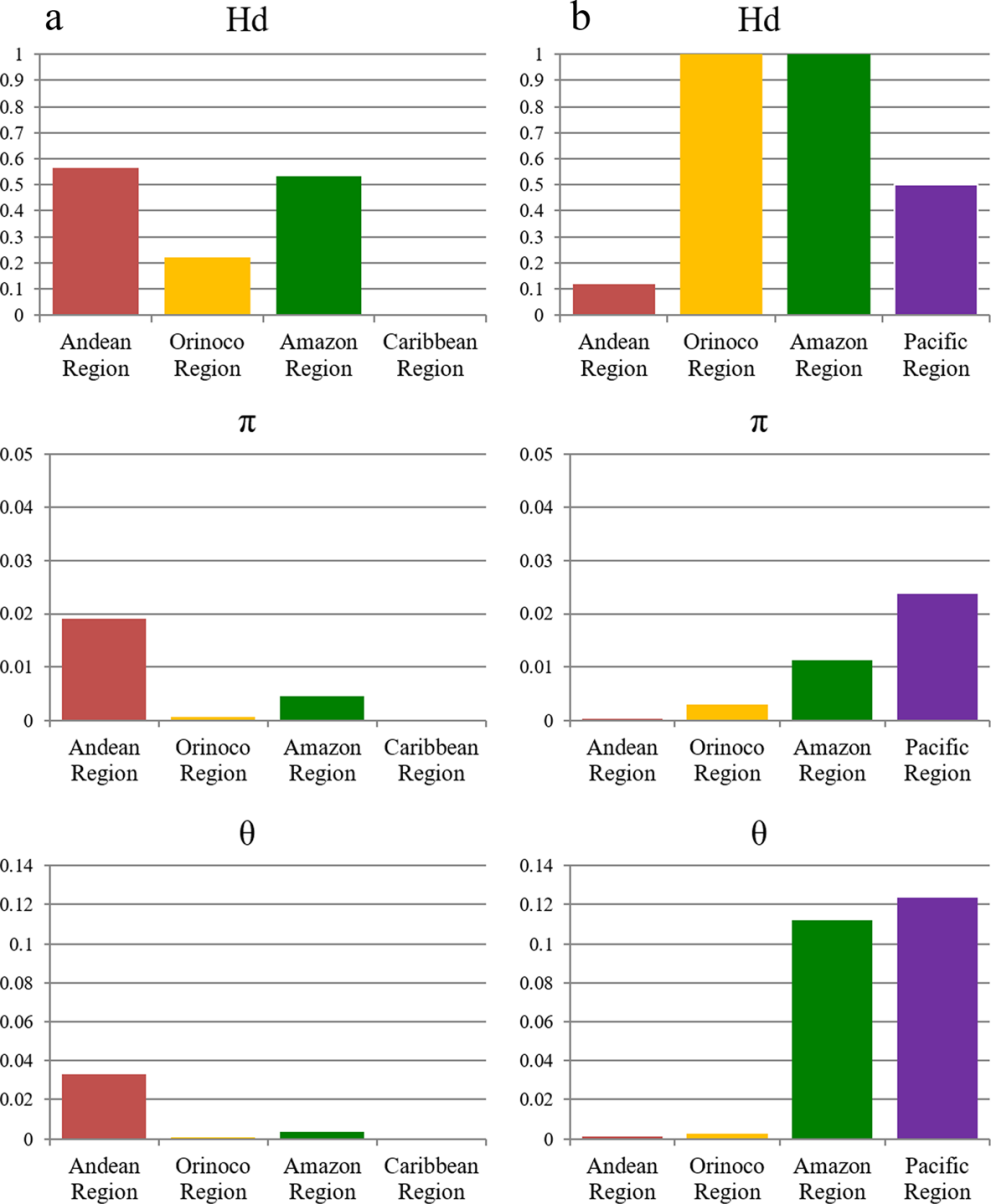
Comparison of genetic diversity indexes (Hd, π and θ) by geographical region for *L. panamensis* (**a**) and *L. braziliensis* (**b**)

When comparing the genetic diversity by geographical region (Fig. 1), it was found that *L. panamensis* had a high degree of haplotype diversity among samples from the regions of the Amazon and Orinoco (Hd = 1 in both regions, *N* = 9 and *N* = 12, respectively) while samples from the Andean region showed less diversity (Hd = 0.118, *N* = 36). When calculating nucleotide diversity indices π and θ the trend remains only in the Andean region, while in the Orinoco region is reversed. Meanwhile, samples of *L. braziliensis* showed greater haplotype diversity in the Andean region (Hd = 0.564, *N* = 36) and Amazon region (Hd = 0.534, *N* = 9), while those from the Caribbean region showed a lack of haplotype diversity (H = 0, *N* = 1). As for nucleotide diversity indices π and θ, the samples from the Andean region showed greater diversity (0.019 and 0.033, respectively), followed by the samples from the Amazon region (0.00451 and 0.00342, respectively).

### Geographical and temporal distribution of DSTs

**Fig. 2:**
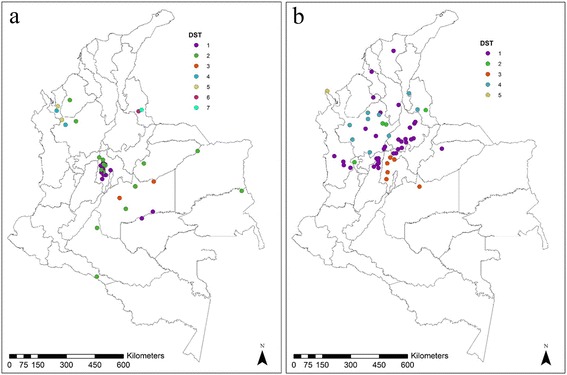
Geographical distribution of diploid sequence types (DSTs) retrieved based on the seven gene markers MLST scheme for *L. braziliensis* (**a**) and *L. panamensis* (**b**)

**Fig. 3:**
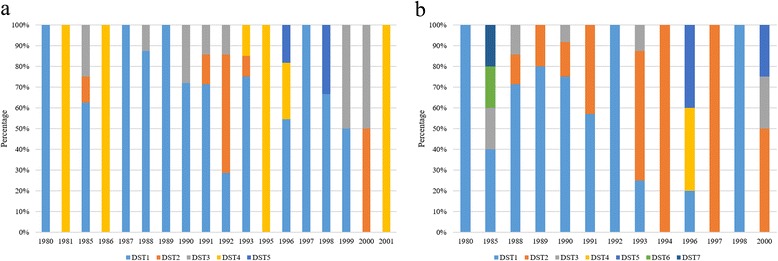
Temporal variation of diploid sequence types (DSTs) retrieved based on the seven gene markers MLST scheme for *L. panamensis* (**a**) and *L. braziliensis* (**b**)

The results from MLSTest showed a total of seven DSTs for *L. braziliensis* and five DSTs for *L. panamensis* (due to the lack of a DST database, the DSTs detected were included in Additional file 2: Tables S1, S2)*.* Based on the DSTs obtained and georeferencing tool data, distribution maps were constructed, showing the distribution of six of the seven DSTs of *L. braziliensis* in the Andean region, with major density in three departments (Antioquia, Cundinamarca and Norte de Santander). DST 2 is uniformly distributed in four of the five regions of Colombia, while the DST 3 is restricted to the Orinoco region; and DSTs 6 and 7 are found only in the department of Norte de Santander (Fig. 2a). In contrast, the distribution of DSTs of *L. panamensis* showed that four of the five DSTs of this species are circulating in the Andean region, with a trend to cluster in the central and western departments of the country. DST 1 was found circulating in the Andean, Caribbean, Orinoco and Pacific regions, while the DST 5 is restricted to the Pacific region (Fig. 2b). We determined the temporal distribution of the DSTs detected (Fig. 3). No association between the DSTs and the year of isolation was observed. Two patterns were depicted: (i) in *L. panamensis*, DST1 was constant across the years but DST 5 only appears in 1996 (Fig. 3a); (ii) in *L. braziliensis* DST 6 and DST 7 only appear in 1985 (Fig. 3b).

### Phylogenetic analyses and clonal complexes

**Fig. 4:**
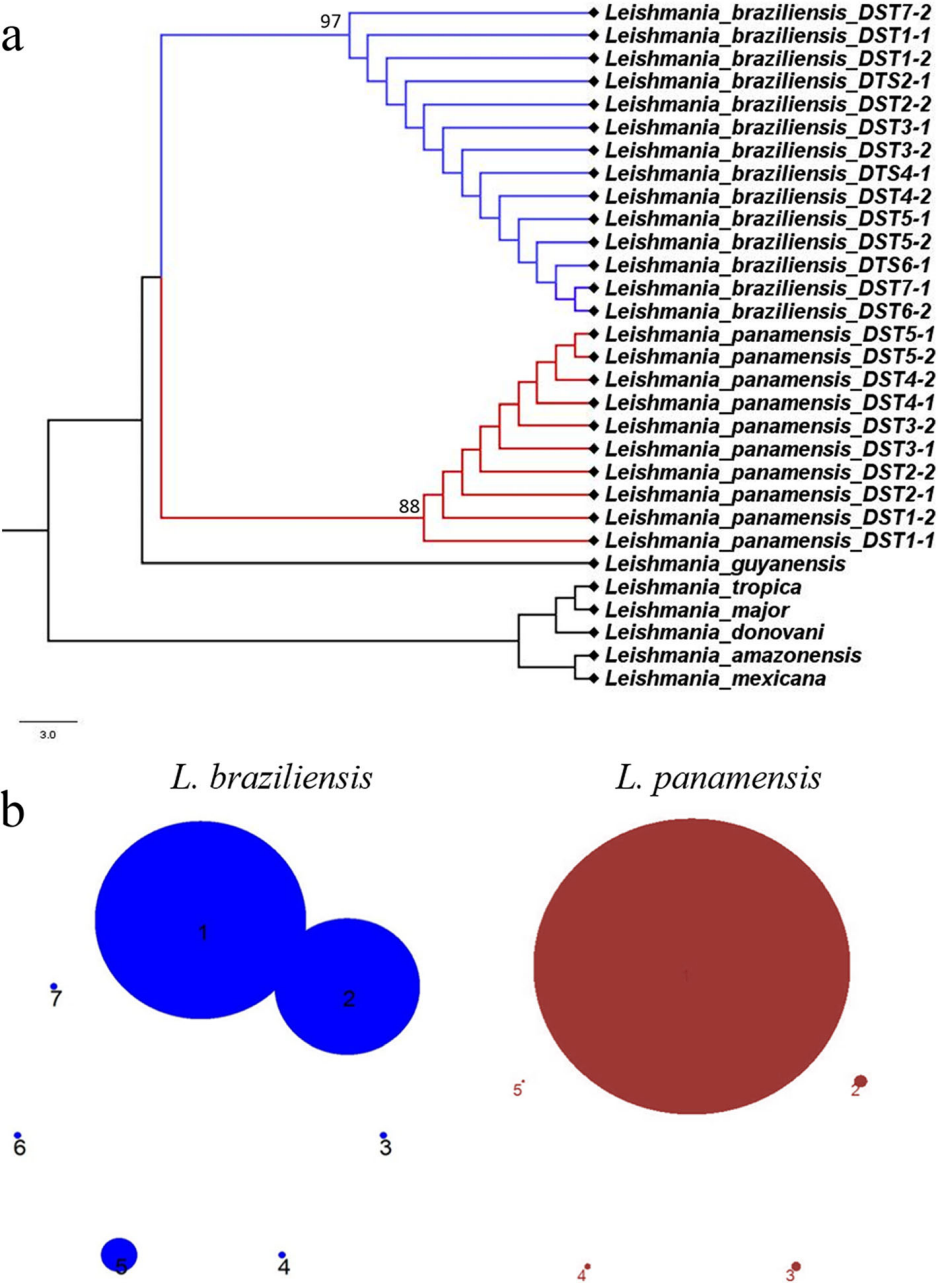
Phylogenetic reconstruction based on the seven gene markers MLST scheme. **a** Maximum likelihood phylogenetic reconstruction cladogram of *L. panamensis* (*red*) and *L. braziliensis* (*blue*) of concatenated sequences of the seven markers employed sowing representatives of the DSTs detected. **b** Clonal complex analysis performed on eBURST showing the number of clonal complexes for *L. braziliensis* (*blue*) and *L. panamensis* (*red*) using the seven gene markers of the MLST scheme

Phylogenetic trees were constructed for each gene fragment including one final concatenated tree (Fig. 4a)*.* The analysis of *L. braziliensis* clonal complex evidenced the presence of two main complexes followed by another five smaller groups. In contrast, *L. panamensis* had only one major clonal complex composed of a predominant genotype with few (4) relatives (Fig. 4b).

### Assesment of clonality *via* allelic plot

**Fig. 5:**
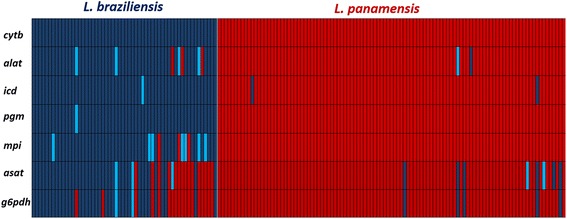
Allelic plot based on the seven gene markers MLST scheme. Allelic plot showing the inheritance of alleles for *L. braziliensis* (*blue*) and *L. panamensis* (*red*) based on phylogenetic reconstruction and considering a true allele with a bootstrap cut-off equal or over 80%. The different shades of blue/red indicate variants of alleles supported by bootstrap (over or equal 80%) within each species studied

Phylogenetic trees were constructed for each gene fragment in order to depict an allelic plot that showed the incongruences in the topologies as a measure of clonality *vs* sexuality (Fig. 5). Overall, the allelic plot for *L. panamensis* showed conservation in the topologies, with few swapping events and exchange of alleles at intra- and interspecies level (Fig. 5). In contrast, *L. braziliensis* showed gross exchange of alleles, mainly presented in the genes encoding *g6pdh* and *asat*. Also, the number of alleles was higher in *asat* than in the other genes tested. In this regard, the genes coding for isocitrate dehydrogenase and phosphoglucomutase in *L. braziliensis* showed conservation in the topologies among the tested samples, while all other genes are events of alleles swapping at intra- and interspecific level with presence of *L. panamensis* alleles. Finally, when calculating the MLGs, a total of 16 MLGs were detected for *L. braziliensis* and 8 MLGs for *L. panamensis*.

## Discussion

Despite the many existing tools for classifying *Leishmania* species, its taxonomy remains contentious. Its complex biology and differing use and interpretation of the biological, biochemical and genetic markers used by the scientific community have not allowed a consensus to be reached, leading some to suggest a need for complete genome sequencing [25–27]. Precisely, this lack of consensus has prevented modification of the current gold standard for typing (MLEE) despite difficulties with its implementation and complex analysis. However, molecular techniques such as MLST are considered useful tools for studies of outbreaks and improving the understanding of the taxonomy of *Leishmania* [25, 26, 28]*.* Despite the benefits of MLST, few studies have been published, most of them focused on the study of Old World *Leishmania* species and only a couple in South America (Brazil and Argentina) [16, 17, 21]*.* To our knowledge, this is the first time MLST has been applied to *Leishmania* spp. in Colombia. Herein, we propose a scheme with seven previously reported different markers for typing the two most prevalent species (*L. panamensis* and *L. braziliensis*) (Table 1) [15–17, 21, 29]. Among the markers employed, the use of *cytb* gene is highlighted due to its specificity and sensitivity including its character of uniparental inheritance [30–33].

*Leishmania panamensis* showed lower diversity than *L. braziliensis*, this was clearly depicted by comparing rates of haplotype diversity of different genes (Table 2). The greatest value of diversity in *L. panamensis* was observed in *pgm* and *alat* markers (Hd: 0.234), which was matched only by the diversity index of *alat* in *L. braziliensis* (Hd: 0.223) (Table 2; Additional file 1: Figure S1). Nevertheless, when comparing the genetic variability of *L. braziliensis* with previously reported data from Argentina and Brazil, the latter exhibit a much greater diversity than in the present study. As shown by Marco et al. [21], haplotype diversity indices for *alat* and *pgm* markers were 0.89 and 0.921, respectively, compared with 0.223 and 0.324 of this study. In this sense, the haplotype diversity values for *g6pdh*, *mpi* and *icd* were closer to those reported by Boite et al. [16] (Table 2; Additional file 1: Figure S1). The differences between the values reported in each study are attributed not only to the fact that genes are different, but also in the study of Marco et al. estimates of genetic diversity were performed based on different species of the subgenus *Viannia*, resulting in an increase in these values of diversity [21]. The other fact is the vast diversity of *L. braziliensis* across South America. Van der Auwera et al. [28] suggested the presence of three different groups within the same species based on the heat-shock protein 70 (HSP70), demonstrating the essential role that geography and ecology can play as a major driver of genetic diversity. However, the genetic variability within *L. braziliensis* will only be resolved when a consensus MLST scheme is applied to isolates across South America. It is noteworthy that *g6pdh* marker showed more diversity in *L. braziliensis* than in *L. panamensis.* This is possibly due to rearrangements present in chromosomes 20 and 34 in *L. braziliensis* where this gene is located [34, 35].

Regarding the geographical distribution of DSTs, it is noted that *L. braziliensis* DST 1 is concentrated in the center of the country (Fig. 2), wheres DTS 2 is widely dispersed. The heterogeneous distribution covers the Caribbean, Andean, Orinoco and Amazon regions. DSTs 4 and 5 were found only in the department of Antioquia. This geographical restriction to a single department was also evident for the DSTs 6 and 7. In contrast, the *L. panamensis* DST 1 is distributed in the Pacific, Andean, Caribbean and Orinoco, while the DST 3 was found in central Colombia (Cundinamarca department). The DSTs 2 and 4 were distributed in departments of the Andean region, while the DST 5 was restricted to the department of Choco (Fig. 2a, b). When these dispersions are compared with the geographical distribution of *Leishmania* vectors in Colombia reported by Ferro et al. [36], we observed that *L. braziliensis* DSTs 6 and 7 are restricted to the department of Norte de Santander overlapping with the distribution of *Pintomyia spinicrassa* in that department; and no other DSTs were found in the other departments (Cundinamarca and Boyacá) [36]. Likewise, a slight overlap of *L. panamensis* DST 3 was observed with the vector *P. ovallesi* in the department of Cundinamarca [36], indicating that this sandfly species might be a potential vector of *L. panamensis* [37]. This suggests a possible selection by the vector of some genetic variants. However, MLST studies in vector isolates are needed to confirm this hypothesis. Regarding the temporal variation, no strict associations were observed based on the DSTs; this can be explained by the strict selection of isolates in this study, a cross-sectional study is required to determine the true temporal distribution of DSTs in Colombia (Fig. 3).

The phylogenetic reconstruction of the concatenated alignment (Fig. 4a) demonstrated the genetic relatedness of the DSTs depicted. The MLST scheme had the power and resolution to readily discriminate *L. braziliensis* and *L. panamensis*, including *L. guyanensis*. Different authors have proposed that *L. guyanensis* and *L. panamensis* might be the same species, but the MLST scheme herein proposed was able to discriminate these two species. However, more *L. guyanensis* isolates are required to fully corroborate this statement. The calculation of the number of clonal complexes confirmed the high diversity of *L. braziliensis* compared to *L. panamensis* (Fig. 3b). Our results reinforce the need to establish a consensus MLST scheme for a proper epidemiological surveillance of cutaneous leishmaniasis, not only in Colombia but at a continental level.

The study of the genetic diversity and population structure of *Leishmania* is complex due to the absence of accurate population genetics tools that correlate with the biology of the protozoan parasites. The type of reproduction of *Leishmania* is controversial, some authors have demonstrated genetic exchange in *Leishmania* [38] but others suggest preponderant clonal evolution [39]. Recently, whole genome sequencing demonstrated the capacity of hybridization in *Leishmania* favouring a sexual propagation theory in protozoan parasites [40]. Our results are consistent with previous results that highlight the ability of *Leishmania* for genetic exchange, where different alleles of *L. panamensis* were observed in the genes of *L. braziliensis* and *vice versa* (Fig. 5). Clear events of intra- an inter-species allelic exchange were observed. This phenomenon of alleles swapping/exchange was more frequently observed in *L. braziliensis*. This has been also demonstrated in Brazil where *L. braziliensis* showed a high level of genetic exchange [41]. Herein, we showed the heightened potential of *Leishmania braziliensis* for genetic exchange and the reduced capacity for genetic exchange observed in *L. panamensis*. Such instances of genetic exchange can impact the distribution of DSTs, since shifting of alleles may generate novel DSTs across time and space. However, that is exactly the benefit of MLST as typing tool in molecular studies since it could be pivotal to understanding emerging strains and outbreaks arising from genetic exchange across endemic regions.

## Conclusions

To our knowledge, this is the first study of MLST in *Leishmania* species from Colombia, which enables a robust and reliable strategy to identify the two most prevalent species in the country. The phylogenetic analyses suggest its applicability to other New World species of *Leishmania,* but a set of well-characterized isolates of these species are required to fulfill this statement. It is also the first study to determine intraspecific genetic diversity of *L. panamensis*, and the high genetic diversity of *L. braziliensis* in Colombia*.* The usefulness of MLST is demonstrated and we encourage the Colombian government to establish a database that can be used for epidemiological surveillance. This will afford establishment of measures for prevention and control; towards early detection of drug resistant and virulent strains that pose substantial threats to the susceptible population that suffer from cutaneous leishmaniasis.

## Additional files

Additional file 1: Figure S1. Comparison of haplotype diversity (Hd) by species. **a** *L. panamensis.* **b** *L. braziliensis*. (PPTX 126 kb)

Additional file 2: Table S1. List of allelic profile by each gene fragment for the assignment of diploid sequence types in *L. braziliensis.* **Table S2**. List of allelic profile by each gene fragment for the assignment of diploid sequence types in *L. panamensis*. (XLSX 14 kb)

## Abbreviations

alt: Alanino-transferase
ast: Aspartate aminotransferase
CL: Cutaneous leishmaniasis
*cytb*: Cytochrome *b*
DST: Diploid sequence type
g6pdh: Glucose 6-phosphate dehydrogenase
icd: Isocitrate dehydrogenase,
INS: Instituto Nacional de Salud
MCL: Mucocutenous leishmaniasis
MLEE: Multilocus enzyme electrophoresis
MLSA: Multilocus sequence analysis
MLST: Multilocus sequence typing
mpi: Mannose phosphate isomerase
pgm: Phosphoglucomutase
VL: Visceral leishmaniasis
