# Supplementary figures and images for: Evaluation of a Multilocus Sequence Typing (MLST) scheme for Leishmania (Viannia) braziliensis and Leishmania (Viannia) panamensis in Colombia
Source: Parasit Vectors. 2017 May 12;10:236. doi: 10.1186/s13071-017-2175-8 (PMC5429539; doi:10.1186/s13071-017-2175-8)

## Slide 1
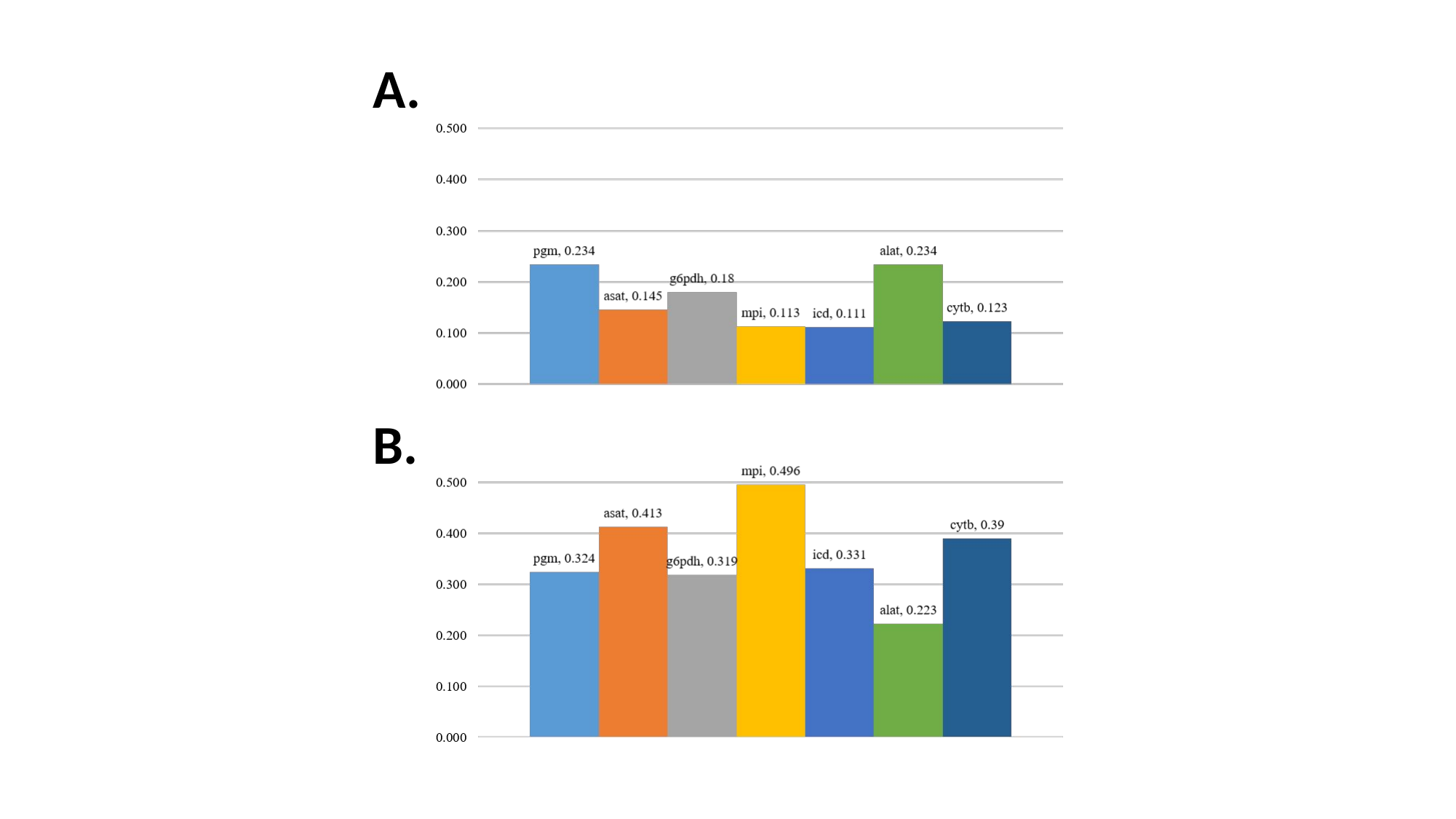

A.
B.

Supplement: Supplementary file 1 — Comparison of haplotype diversity (Hd) by species. a L. panamensis. b L. braziliensis. (PPTX 126 kb) [file 13071_2017_2175_MOESM1_ESM.pptx]
